# Interest of Bone Histomorphometry in Bone Pathophysiology Investigation: Foundation, Present, and Future

**DOI:** 10.3389/fendo.2022.907914

**Published:** 2022-07-28

**Authors:** Pascale Chavassieux, Roland Chapurlat

**Affiliations:** Université de Lyon, INSERM, UMR 1033, Lyon, France

**Keywords:** bone biopsy, histomorphometry, bone disease, modeling, remodeling, mechanism of action of treatment

## Abstract

Despite the development of non-invasive methods, bone histomorphometry remains the only method to analyze bone at the tissue and cell levels. Quantitative analysis of transiliac bone sections requires strict methodologic conditions but since its foundation more 60 years ago, this methodology has progressed. Our purpose was to review the evolution of bone histomorphometry over the years and its contribution to the knowledge of bone tissue metabolism under normal and pathological conditions and the understanding of the action mechanisms of therapeutic drugs in humans. The two main applications of bone histomorphometry are the diagnosis of bone diseases and research. It is warranted for the diagnosis of mineralization defects as in osteomalacia, of other causes of osteoporosis as bone mastocytosis, or the classification of renal osteodystrophy. Bone biopsies are required in clinical trials to evaluate the safety and mechanism of action of new therapeutic agents and were applied to anti-osteoporotic agents such as bisphosphonates and denosumab, an anti-RANKL, which induces a marked reduction of the bone turnover with a consequent elongation of the mineralization period. In contrast, an increased bone turnover with an extension of the formation site is observed with teriparatide. Romosozumab, an anti-sclerostin, has a dual effect with an early increased formation and reduced resorption. Bone histomorphometric studies allow us to understand the mechanism of coupling between formation and resorption and to evaluate the respective role of bone modeling and remodeling. The adaptation of new image analysis techniques will help bone biopsy analysis in the future.

## Introduction

Despite the development of non-invasive methods as bone densitometry, biochemical markers, and quantitative computed tomography, bone histomorphometry remains the only method for the study of bone at the tissue and cellular levels. This method enables measurements at intermediary levels of bone organization, i.e., the osteon in cortical bone and the bone structural unit (BSU) in cancellous bone. Bone histomorphometry consists of counting the cells and measuring the bone tissue components. The field of bone histomorphometry appeared in the early 1960s and markedly progressed over the following decades. The sequence of activation-resorption-formation and the basic multicellular unit (BMU) initially described by Frost (1–3) led to defining the concept of bone remodeling. Each remodeling site gives rise to a BSU (4) which constitutes bone tissue. In addition to the measurements of static parameters which give an imprint of previous remodeling events, the introduction of double labeling with tetracycline, which is laid down at the mineralization front, provides a time dimension (3). The methods used for the measurements on histological sections are based on Delesse’s principle (5) which allows the deduction of 3D parameters from 2D measurements. A variance in the bone histomorphometric measurements may be observed which results from the methodology used and the inter-observer variation. The main methodological factors are the sampling, staining procedures, and the measuring methods. For these reasons bone histomorphometry requires strict and standardized methodological conditions. The diagnosis of the majority of bone diseases is based on clinical, radiological, and biochemical examinations but a bone biopsy can be required to evaluate a mineralization defect, to determine the form of renal osteodystrophy, or to understand a non-response to treatment. Bone histomorphometry is the only method for the analysis of the pathophysiology of bone diseases and is mandatory for the safety and mechanism of action of new drug at the tissue and cellular levels. It also allows to investigate and understand the mechanism of coupling between resorption and formation during the remodeling process and the role of the modeling. Our purpose was to review the evolution of bone histomorphometry across the years including the improvement in the methods, the development of new parameters, the conditions required for a suitable analysis, the knowledge in bone tissue metabolism under normal and pathological conditions, and the understanding of the mechanisms of action of therapeutic drugs in humans. We also considered future applications.

## Bone Biopsy

A bone biopsy is necessary to obtain a bone sample.

### Procedure

A horizontal transiliac bone biopsy is the preferred method (6). It gives a sample with two cortices and a sufficient amount of spongy bone (7), in contrast to the vertical biopsy which provides only one cortex (8). Transiliac bone biopsy must be taken at the standard site i.e., 2 cm below the summit of the iliac crest and 2 cm behind the antero-superior iliac spine. It requires local anesthesia of both the internal and external periosteum. Bone biopsy is safe and generally well-tolerated. The incidence of complications reported is low, at 0.52% with mainly hematomas, pain, transient femoral neuropathy, and skin infection (9). If a follow-up biopsy must be performed, it must be done on the opposite side to avoid the prior callus. Several types of trephines for bone biopsy have been used (7). The 2 mm diameter Jamshidi needle (10) does not allow for suitable evaluation of bone turnover and mineralization (11). Trephines with a diameter of 5 mm were responsible for a large sampling variation (12, 13). A trephine of 7.5 mm inner diameter, as Bordier trephine modified by Meunier, provides a transiliac sample with two cortices and a sufficient amount of spongy bone suitable for histomorphometry. An electrically driven trephine has also been proposed (14). The trephine teeth must be perfectly sharpened to avoid compression artifacts and fracture of the specimen which may compromise the analysis. The experience of the operator is another factor that contributes to the quality of the sample (9).

### *In Vivo* Tetracycline Labeling

**Figure 1:**
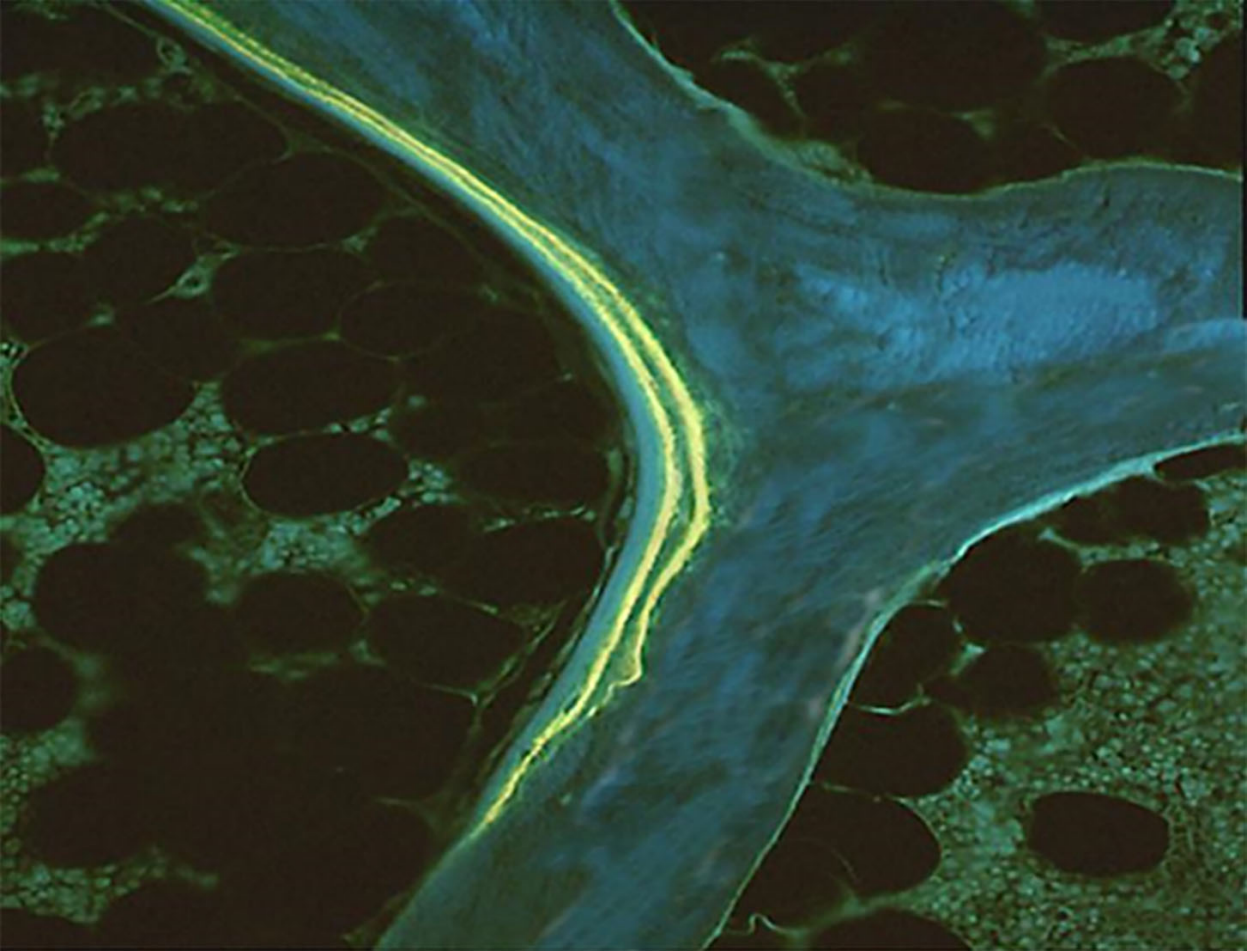
Double tetracycline labels. Unstained section under ultraviolet light; x100.

Prior to biopsy, the administration of fluorochromes allows the measurement of dynamic parameters of bone formation and consequently under specific conditions, the assessment of bone turnover. There are several fluorochrome regimens (15, 16) but tetracycline is a safe tissue marker (3). Tetracycline incorporates into new bone at the time of the mineralization and remains as long as the bone is not resorbed (17). Alizarin, another tissue marker, inhibits the bone formation and must not be used in humans (18). Its use must be limited to animal studies and given just before the biopsy. Different tetracycline regimens have been used and the choice mainly depends on the availability in the center. They are equivalent despite some differences observed with respect to their uptake by the mineralizing bone. Parfitt et al. (19) showed that the extent of surfaces labeled with demethylchlortetracycline were higher than with oxytetracycline. The difference may be explained by a lower blood level, a shorter half-life, and a lower affinity for binding to the crystal for oxytetracycline than demethylchlortetracycline (20). Different schedules of double labelings are used but the current procedure is to administer tetracycline for two sequences of 2 days 10 days apart, with the biopsy being performed 3-5 days after. Tetracycline deposited, along the calcification front in bone, appears as two distinct lines visualized on unstained bone sections under ultraviolet light (3) (**Figure 1**). More recently, another labeling schedule has been proposed (21). It is based on a quadruple labeling with a first double labeling at baseline and a second one a few months later (≤ 3 months). This method allows longitudinal analysis of short-term changes of bone formation in one single biopsy. This technique presents two advantages over paired biopsies: only one sample is collected in each patient and each serves as his/her own pre-treatment control, eliminating problems due to the inter-individual variability in histomorphometric variables (22). The second labelling must be administered less than three months after the first one to avoid the resorption of the first set of labels and to allow the calculation of parameters expressed with bone surface as referent because bone surfaces used for the calculation of the baseline parameters are those measured at the time of biopsy. The potential variation of the extent of bone surface after 1-2 months is very low and markedly lower than the inter-individual variation.

### Histological Techniques

#### Fixation and Embedding

Bone samples must be processed without prior decalcification. Upon the collection, the sample must be placed in fixative, methanol, 70% ethanol, or 10% phosphate-buffered formalin (pH 7). However, alcoholic fixatives are recommended to preserve the tetracycline labels. The embedding compound must be as hard as the calcified bone to preserve the integrity of the bone architecture during sectioning. Different embedding materials have been used such as Epon or Bioplastic but methyl or glycol methacrylate are the most convenient (23, 24). In addition, these plastics may be dissolved before staining but this procedure may damage the bone architecture. As polymerization of methacrylates is an exothermic reaction, enzyme activity and antigenic characters are lost by high temperatures. Several authors have reported modifications of the conventional methacrylate techniques performed at low temperatures allowing the preservation of enzymes activities (25–28)

#### Sectioning

Sections, 5 to 20 µm thick, are cut using special microtomes equipped with tungsten carbide-edged knives or with diamond or glass knives (24). Sections can be also obtained by polishing. These knifes must be perfectly sharpened to obtain sections of good quality. The block is oriented so that the cortices are perpendicular to the knife edge. It is recommended to obtain two or three sets of sections in the central part of the sample, separated by 200-300 µm to avoid replicate sampling of a single surface event (29–31).

#### Staining

**Figure 2:**
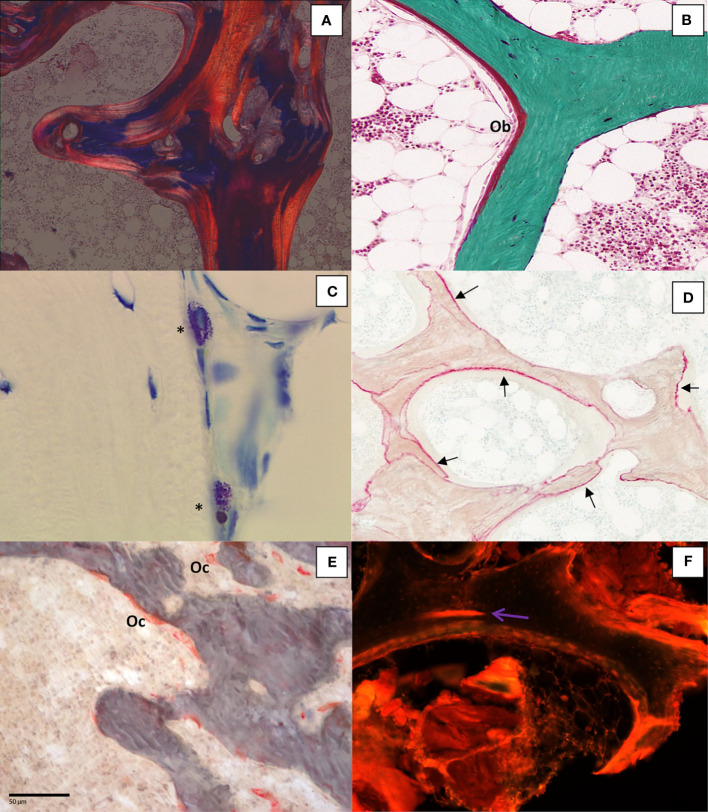
**(A)** Bone section stained with solochrome cyanin R under polarized light showing the lamellar texture of bone tissue (x100); **(B)** Goldner trichrome differentiates mineralized bone in green and osteoid seam covered by osteoblasts (Ob) in red (x200); **(C)** Mast cells (*) in bone marrow characterized by the presence of metachromatic granules in the cytoplasm after toluidin blue pH 2.6 staining (x400); **(D)** Aluminon staining showing the aluminum deposition (→) along the calcification front (x100); **(E)**.TRAP staining of osteoclasts (Oc) (x200); **(F)**: Microcrack (→) stained after bulk staining with xylenol orange (x200).

The initial objectives of stain were to unequivocally differentiate osteoid and mineralized bone and to identify cells. Von Kossa was initially recognized as the reference method (24, 32) but it was not ideal for cellular details. Villanueva osteochrome and tetrachrome have also been used (33) and a modification of the osteochrome was described and allowed the simultaneous assessment of tetracycline and osteoid seams (34). Solochrome Cyanin R (35) is very good for staining osteoid and for observing the bone texture under polarized light (**Figure 2A**) but the manufacturer ceased production several years ago. A stain for osteoid tissue in the fresh, unembedded bone sample has also been reported (36). Toluidine blue stains the calcification front as a granular metachromatic dark line but the most suitable way to identify the calcification front is the use of tetracycline labeling (37). Today, Goldner’s trichrome is the most widely used (38–40). It allows a clear identification of osteoid and bone cells with a sufficient contrast for analyses with image analyzers (**Figure 2B**).

Besides the assessment of bone structure and remodeling, specific staining techniques are applied for either diagnosis or research purposes. The diagnosis of a bone mastocytosis is confirmed by the count of mast cells on histological bone sections. Mast cells are identified on sections stained with May-Grünwald-Giemsa, acridine orange or toluidine blue pH 2.6. Based on the principles of metachromasia, mast cells granules are stained purple by toluidine blue pH 2.6 (41, 42) (**Figure 2C**). The contamination of dialysis with aluminum led to the accumulation in patients with chronic renal failure undergoing haemodialysis (43). Aluminum accumulation has also been reported after regular consumption of aluminum-based antiacids (44). Overload of aluminum was shown to be responsible for vitamin D resistant osteomalacia. Measurement of serum aluminum was not sufficiently discriminant for assessment of aluminum intoxication in these patients. Bone aluminum can be shown by a specific histochemical staining with aurine tricarboxylic acid (aluminon staining). Aluminum deposits appear as a deep pink to red line on the mineralized surfaces (**Figure 2D**) (45–47). Other stainings have been reported such as acid solochrome azurine (Mordant blue) or a technique described by Walton et al. (48) where aluminum forms a complex with the carboxylate group of the phoxine dye.

Osteoclasts are known to have a high tartrate-resistant acid phosphatase (TRAP) content. A staining procedure allows specific identification of osteoclastic acid phosphatase. This technique has been shown to stain only osteoclastic acid phosphatase enzymes and not induce artifacts (49). As previously mentioned, it requires a prior embedding process at low temperature to not alter the enzyme activity. TRAP is located in lysosomes and reflects the osteoclasts function (50, 51) (**Figure 2E**).

Bone microdamage is essential in the assessment of bone quality because its accumulation, due to fatigue loading, may lead to fracture (52). The method allowing the detection of microdamage in bone must distinguish *in vitro* microdamage caused by the histological process from the naturally occuring one. This requires making bulk staining, i.e., staining before embedding and sectioning. The first technique used basic fuchsin (53). However basic fuchsin is not specific because it binds to exposed collagen but does not bind to the mineral phase (54). Different dyes such as oxytetracycline, xylenol orange, green calcein, and calcein blue bind microcracks (55). They fluoresce at different wavelengths and colors and can be sequentially used to follow the progression of microcracks *in vitro* (56). Some dye, e.g., xylenol orange, do not hamper the reading of tetracycline labeling (57) (**Figure 2F**).

Several years ago, techniques staining the cement lines were described. They were based on the use of either gallocyanin associated with Villanueva which stained cement lines in blue or purple (40) or toluidine blue dissolved in 0.1% formic acid (58) which stained cement lines in dark blue. More recently, the interest of analyzing the cement lines has been raised to identify remodeling and modeling-based formation. To date, these two phenomena were usually differentiated by the morphological aspect of these cement lines which, by definition, are crenated after remodeling and smooth after modeling. Recently, an immunostaining of osteopontin has been reported, which differentiates osteopontin-rich smooth cement lines formed by modeling from osteopontin-poor scalloped cement lines generated by remodeling process (59).

## Histomorphometry

### Stereological Principles

Bone histomorphometry consists in the measurement of a three-dimensional structure on a two-dimensional thin section. Basic stereological theorems based on the Delesse principle (5) report the mathematical proofs that a volume can be extrapolated from the area of its profiles and there is a relationship between its surface and the profile of its boundary length (60–64). However, the prerequisite of most stereologic theorems is that the structure is isotropic, i.e., dispersed and randomly oriented in the space (65, 66). This condition is not always fulfilled in bone, as the microarchitecture is mainly determined by the mechanical forces, except in iliac bone. In cortical bone, the main axis of the Haversian canals is parallel to the longitudinal axis of the diaphyseal cortex (67) and cancellous bone consists of trabeculae more or less oriented (68). Random, but anisotropic, sections obtained with a fix axis and called “vertical sections”, can provide an unbiased estimation (69). This sampling procedure has been applied to bone by Vesterby et al. (70) where the vertical axis is parallel to the cylindrical core surface, the sections are obtained with a random rotation around the axis, and a cycloid test grid is used.

### Measuring Methods

#### Validation

Different methods have been used for the quantification of the bone components. The first step before using a method is its validation, i.e., the evaluation of accuracy, reproducibility, and linearity.

The accuracy depends on the lack of bias and the precision. Measurements are biased when they systematically differ from the true value. The precision is given by the narrow dispersion of measurements (66). The reproducibility reflects the variation between repeated measurements performed either by the same observer (intra-observer variability) or by different observers having the same experience (inter-observer variability). The linearity assesses the relationship between the measures and the true values for a large range of values. These evaluations are performed by using standard micrometers for image analyzers and standard bone sections and are applied for the measurements of distance, length, and area.

#### Methods

Initially, the measurements were performed with a manual point-counting method using integrative eyepieces which consisted of projecting straight or semicircle parallele lines and points on the microscopic field (66). More recently, this method has been almost abandoned and replaced by interactive computerized analyzers.

Semi-automatic systems are composed of a microscope equipped with a drawing tube, a digitizing tablet, and a cursor. The image of the cursor light is projected on the microscopic field and the measurement is performed by tracing the structure to be measured on the digitizing tablet. The *x* and *y* coordinates of each point of the tablet are integrated by a computer which gives the results according to the previously selected program package (71–73).

With the development of computerized technique, the automatic method image analyzer processes, developed 50 years ago (74, 75), have been equipped with cameras whose imaging system uses separate charge-coupled devices (3CCD) which capture the image of the microscopic field. The image recorded in the computer is displayed on a high-definition video monitor (76–80). Bone tissue is detected according to a selected color threshold and parameters of bone structure and microarchitecture are automatically provided. In addition, interactive measurements of parameters reflecting the bone turnover can be also obtained (80, 81). The equipment now associates automatic and semi-automatic systems. The measurements of the different components of the bone tissue can be performed on a live image and traced directly on the computer screen with *x-y* positions of the enhanced live overlay linked to the motorized *x-y* stage. In addition, live image stitching makes it possible to obtain large mosaic-images that offer the advantage of a wide field and very high resolution.

### Histomorphometric Parameters

#### Terminology

A standardization of the nomenclature for histomophometric parameters was initially established by the ASBMR committee in 1987 (82) and updated in 2013 (31). The objective of this nomenclature is to clarify the terminology used in the past and gives the abbreviations which must be used in all bone histomorphometric studies. The principle is to express all data by using the same format, i.e., Source-Measurement/Referent where the source is the region (cortical (Ct), cancellous (Cn), endocortical (Ec), periosteal (Ps) bone, or total core (Tt)). All measurements must be expressed as an index of the amount of tissue analyzed which are mainly bone surface (BS), bone (BV) volume, or tissue (TV) volume of the region analyzed. Only the measurements of distance, i.e., width, can be given without referent. The data can be expressed in two-dimensional, e.g., width (Wi), perimeter (Pm) and area (Ar), or three-dimensional e.g., thickness (Th), surface (S) and volume (V), terminology, but only one type of terminology can be used in an article.

#### Parameters of Bone Resorption

The main problem in assessing bone resorption is to analyze a structure which has disappeared (**Table 1**). Eroded surfaces are identified by their irregular and crenated aspects with the presence of osteoclasts but resorption cavities may not be deep and without the presence of osteoclast. The identification of eroded lamellae under polarized light may help the characterization of lacunae (83). As previously mentioned, osteoclasts can be identified by the presence of tartrate resistant acid phosphatase. Erosion depth reflects the activity of osteoclasts. Different procedures to assess the erosion depth have been reported. The first one was based on the inverse relationship between erosion depth and the interstitial width, i.e., the distance between two BSU situated on opposite sides of a trabecula (84). However, this relationship is influenced by any changes of the wall width and trabecular thickness (85, 86). Other authors evaluated erosion depth by counting the number of eroded lamellae and measuring the thickness of lamellae (87). In addition, these authors separated eroded cavities according to the presence of osteoclasts, mononuclear, or preosteoblastic cells related to the stages of resorptive phase. This method depends on the ability to suitably identify these different cell types, only on their morphology. This method has never been used by other groups. A computerized method (88) and an interactive reconstruction approach (80) have been used to reconstruct the bone surface before the onset of the resorption. This method allows only an estimation of the erosion depth (mean and maximum) and volume because measurements are performed independently of the resorption stage, including resorption cavities not totally achieved. Despite an underestimation of the erosion depth, a correlation has been found with deoxypyridinoline, a marker of the bone resorption (80). A true final erosion depth has been obtained with a similar method applied only on cavities covered with a thin layer of osteoid, ensuring that the resorption has ended (89) but with this method there is a limit to the number of measurements that can be done.

**Table 1:** Measured parameters reflecting the bone remodeling and mineralization.

Parameters	Abbreviations	Units
Osteoide surface	OS/BS	%
Osteoid volume	OV/BV	%
Osteoid thickness	O.Th	µm
Eroded surface	ES/BS	%
Erosion depth	E.De	µm
Eroded volume	EV/BV	%
Osteoclast surface	Oc.S/BS	%
Osteoclast number	Oc.N/BS	#/mm
Single labeled surface	sLS/BS	%
Double labeled surface	dLS/BS	%
Inter label distance	Ir.L.Th	µm

BS , bone surface; BV, bone volume.

#### Parameters of Bone Formation

Bone formation includes two stages, the first one is the matrix apposition by the osteoblasts followed, after a delay, by the mineralization process. They can be quantified by the static parameters of bone formation (**Table 1**) and by the dynamic parameters derived from the measurement of tetracycline labels (90) (**Table 2**). Activation frequency (Ac.f; #/year) is the probability that a new remodeling will appear at any point on the bone surface and represents the birthrate of a new remodeling site (91). Activation frequency is derived from parameters of formation while the activation of a remodeling results in resorption. A coupling between resorption and formation is required for a valid interpretation of activation frequency.

**Table 2:** Derived histomorphometric parameters of bone remodeling and mineralization.

Parameters	Abbreviations	Formulae	Units
Mineralization rate	MAR	Ir.L.Th/Ir.L.t	µm/day
Mineralizing surface	MS/BS	((dLS+ sLS/2)/BS)	%
Bone formation rate	BFR/BS	MAR * MS/BS	µm^3^/µm^2^/day
Activation frequency	Ac.f	(BFR/BS)/W.Th	#/yr
Adjusted appositional rate	Aj.AR	MAR/(MS/OS)	µm/day
Mineralization lag time	Mlt	O.Th/Aj.AR	days
Osteoid maturation time	Omt	O.Th/MAR	days
Formation period	FP	W.Th/Aj.AR	days
Active formation period	FPa+	W.Th/MAR	days
Quiescent period	QP	FP *(QS/OS)	days
Resorption period	Rs.P	FP * (Oc.S/OS)	days
Reversal period	Rv.S	FP * (ES-Oc.S/OS)	days

Ir.L.t,  inter-labels time; W.Th, wall thickness; QS, quiescent surface.

#### Parameters of Bone Structure

The bone mass and microarchitecture are major determinants of the bone strength and the measurements of cortical thickness and cancellous bone volume were two of the first parameters assessed (**Table 3**). In recent years, non-invasive imaging methods such as quantitative microcomputed tomography (microCT) or high-resolution peripheral quantitative computed tomography (HR-pQCT) provide three-dimensional structural parameters at peripheral sites (hip and spine) but these techniques detect only mineralized tissue. MicroCT can be also applied to bone samples allowing the measurement of 3-dimensional microarchitecture parameters. Most of the parameters obtained by non-invasive methods correlate with those measured by histomorphometry (93). However, due to the difference in resolution (80 µm for HR-pQCT and less 5 µm for histomorphometry), differences may be observed for a few parameters such as cortical porosity (94), as pores with a small diameter cannot be detected by microCT in contrast to histomorphometry.

**Table 3:** Histomorphometric parameters of bone structure and microarchitecture.

Parameters	Abbreviations	Units
*Measured parameters*		
Cortical thickness	Ct.Th	µm
Cortical porosity	Ct.Po	%
Bone volume	BV/TV	%
Surface density	BS/BV	%
Wall thickness	W.Th	µm
*Derived parameters*		
Trabecular thickness*	Tb.Th	µm
Trabecular number	Tb.N	#/mm^2^
Trabecular separation	Tb.Sp	µm

*An indirect evaluation of trabecular thickness can be calculated from area and perimeter which is based on a plate model (92).

The trabecular connectivity is another determinant of the bone strength. Parameters have been described including the strut analysis (76) and the evaluation of the spatial distribution of trabeculae, their connectivity, and complexity (42, 81, 95–97). Trabecular bone pattern factor (TBPf; #/mm) and Euler number provide an estimation of the trabecular bone connectivity and complexity and have been shown to correlate with bone strength independently to the bone quantity (98). Fractal dimension (FD) reflects the degree of complexity (99–102). The fractal dimension can be assessed on images acquired by radiography, microCT, and on histological sections. A correlation between the fractal dimension and mechanical properties of bone have been reported (103–105).

These microarchitecture parameters provide different and complementary informations on the trabecular network. They have been used until the development of microCT, and several microCT outcomes were derived from them, e.g., connectivity was derived from the Euler number (106).

### Reproducibility of Bone Histomorphometry

Even when suitable methods have been applied, several factors can influence measurements as variations in the results may be observed between laboratories and few centers around the world have the equipment and expertise (107). For these reasons, the methods must be precisely described in manuscripts reporting histomorphometric studies. The main causes of these variations may be due to the sampling, laboratory processing, and measuring methods.

#### Inter-Sample Variation

Several studies investigated different locations of iliac biopsies as the iliac crest is not perfectly isotropic and the trabecular organization can vary between sites within the ilium (65). Even if some differences are observed between the left and right side (108–110) or between different localizations within iliac crest, they were not significant in a group of patients (109–113). However, in a single patient having repeated biopsies, these variations must be considered to affirm the efficacy of treatment (109, 110, 113) and to design the patient group size in clinical trials to detect significant differences when repeated biopsies are conducted (114). Within one core, the variance decreases when the number of microscopic fields and sections increase (115, 116). This points out the recommendation to use a trephine with a 7.5 mm inner diameter and to measure a total tissue area of at least 30 mm^2^ (30, 31) on sections cut in 2-3 plans separated by 200-300 µm.

#### Laboratory Processing

Different staining dyes have been applied to differentiate osteoid and mineralized tissue. Despite significant correlations, some differences were reported between Masson trichrome and toluidine blue (111) or solochrome cyanin and trichrome (117). The measurement of distance may be also influenced by the thickness of the sections (66, 118) and the section obliquity during sectioning. They result in an apparent profile of the structure different from the true value and this variation depends on the thickness of the section and the angle from an ideal perpendicular section. For cortical sections, the projection error is negligible, regardless of the section thickness. For the other thickness measurements, the obliquity can be corrected by multiplying the measured values by π/4 (66).

#### Measuring Methods

Previous studies reported a good correlation between parameters measured by a manual point counting method and computerized image analyzers (72, 73) with coefficients of correlation ranging from 0.90 to 0.98 (73). The variation between methods was found higher than the inter-observer variation (119). However, some variations may exist between different equipments requiring a validation procedure when a new equipment is acquired. It also points out the potential problems associated with the use of control data from other laboratories using different methods (119).

#### Inter-Observer Variation

Inter-observer variation may be important when measurements are performed by different laboratories using different techniques such as staining, magnification or measuring methods (119–121). It shows that it is difficult for one group to refer to the normal range established by another group without a previous cross-calibration. The experience of the observer is also a major factor of inter-observer variation (120). However, when measurements are performed by two experienced observers from the same group, inter-observer variation is lower than 6% (73, 115).

## Bone Biopsy as a Diagnosis Tool

**Figure 3:**
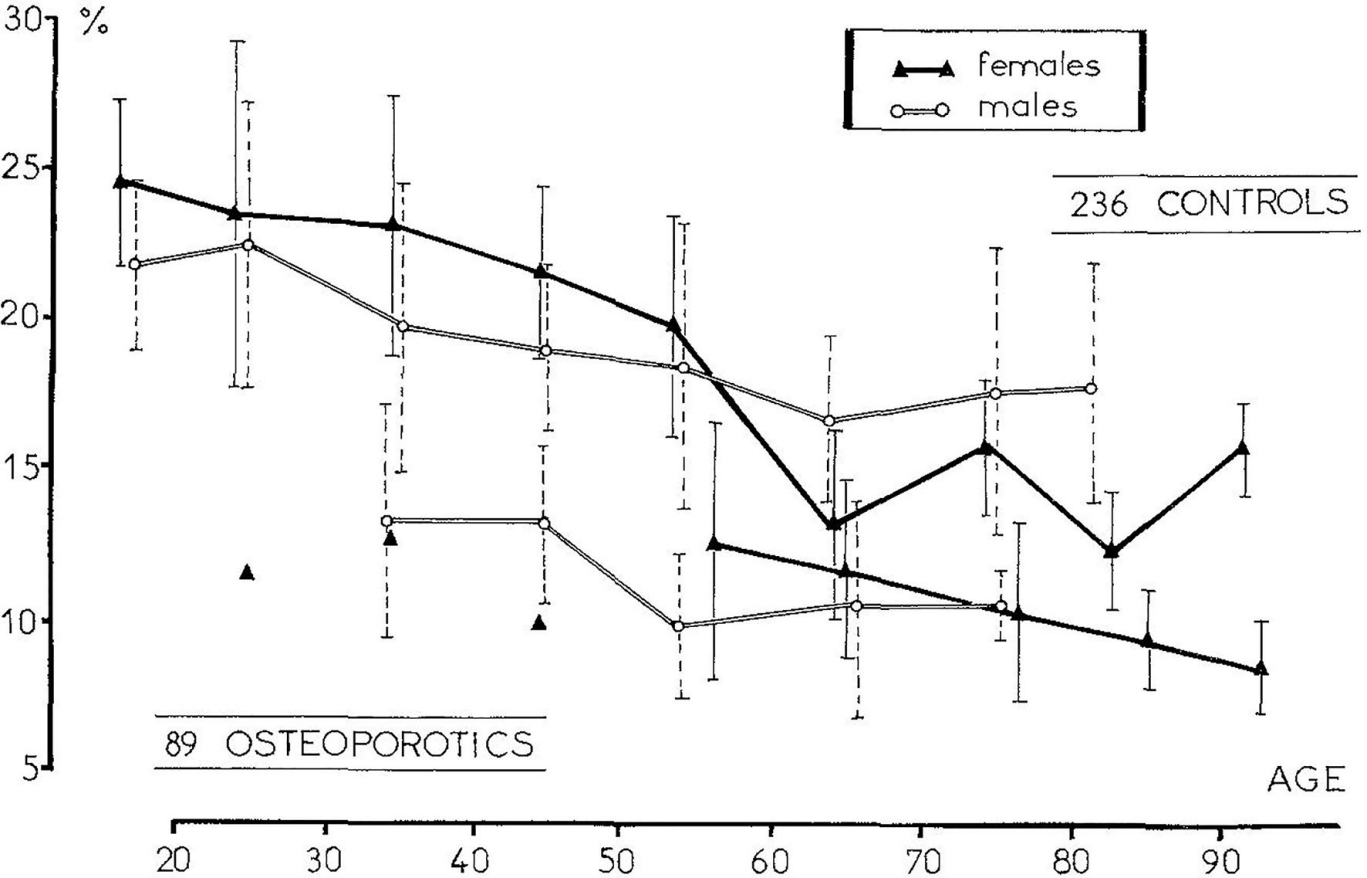
The measurement of the trabecular bone volume in osteoporotic patients with one vertebral fracture allowed to define a “vertebral fracture threshold” at 11% [From Meunier et al. (126)].

Initially, transiliac bone biopsy was widely used for the diagnosis of metabolic bone diseases, mainly osteoporosis. The main question was whether the iliac crest represents the entire skeleton, as the iliac crest is an unloading site without a high fracture risk. Relationships between the iliac bone and vertebrae were reported for the amount of bone and strength (122–125). A cancellous bone volume per tissue volume (Cn-BV/TV) lower than 11% was defined as the “vertebral fracture threshold” in osteoporotic patients (**Figure 3**) (126) and was used for many years as the reference value for the diagnosis of osteoporosis. Despite differences in microarchitecture and turnover between the iliac crest and the other skeletal sites, especially the sites prone to fracture as vertebra and femoral neck (127), significant correlations were found (95, 128) and age-related changes were observed in all sites (129). During the 1980s, the development of non-invasive methods, such as bone densitometry, allowed for the diagnosis of osteoporosis. However, bone histomorphometry remains the only method that allows the analysis of bone at the intermediary level of organization, i.e., the osteon in cortical bone and the bone structural unit in cancellous bone and to assess some key features, e.g., the mineralization or the bone texture.

Normative data for histomorphometric parameters, according to age and sex, have been established in most of histomorphometry laboratories (112, 130–139) which allows to conclude an abnormal bone remodeling, mineralization, and structure (140).

Presently, the main indications of bone biopsy are the diagnosis of osteomalacia, the characterization of renal osteodystrophy, or the investigation of bone fragility not responding to osteoporotic treatment to exclude a bone mastocytosis or any other rare causes of secondary osteoporosis. Beside the quantitative analysis, qualitative observation of bone sections provides additional information as a normal lamellar or woven texture, the presence of microcallus, mineralization defects, and any abnormalities in the bone marrow.

### Osteomalacia

**Figure 4:**
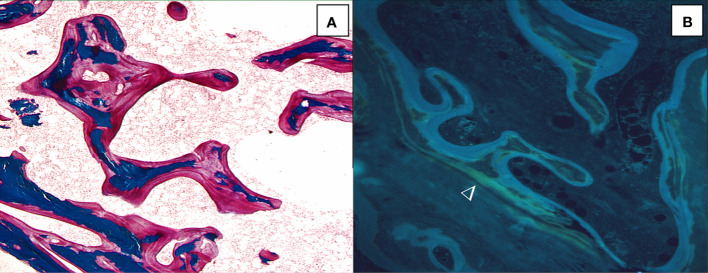
Osteomalacia is characterized by thick and extended osteoid seams (**A**, solochrome cyanin R, x100) and diffuse tetracycline labels (►) (**B**, unstained section under ultraviolet ligth; x100).

Osteomalacia can be diagnosed on biochemical and radiological signs but bone biopsy is the only means to diagnose osteomalacia when these signs are not evident. Osteomalacia is characterized by osteoid accumulation with a thickening of osteoid seams associated with a decreased mineralization rate and an elongation of the mineralization lag time (141) (**Figure 4**). Double labels may be undetectable and appear diffused. A decreased mineralization rate alone is not specific of osteomalacia, it may be a sign of osteoblast activity diminution with a reduced matrix apposition. An increase in osteoid thickness with a normal mineralization rate may reflect an increased apposition rate (32). Osteomalacia is due to vitamin D deficiency but can also complicate chronic renal failure and be a consequence of aluminum intoxication in hemodialysis patients (142–144). Osteomalacia can also occur as a complication of previous gastric surgery, coeliac disease, or long-term parenteral nutrition (145). Recently, mineralization defects in jaw biopsies have been reported in bisphosphonate-treated patients suffering from an osteonecrosis of the jaw. Osteomalacia cannot be considered as the cause of osteonecrosis but may contribute to its development (146, 147).

### Renal Osteodystrophy

**Figure 5:**
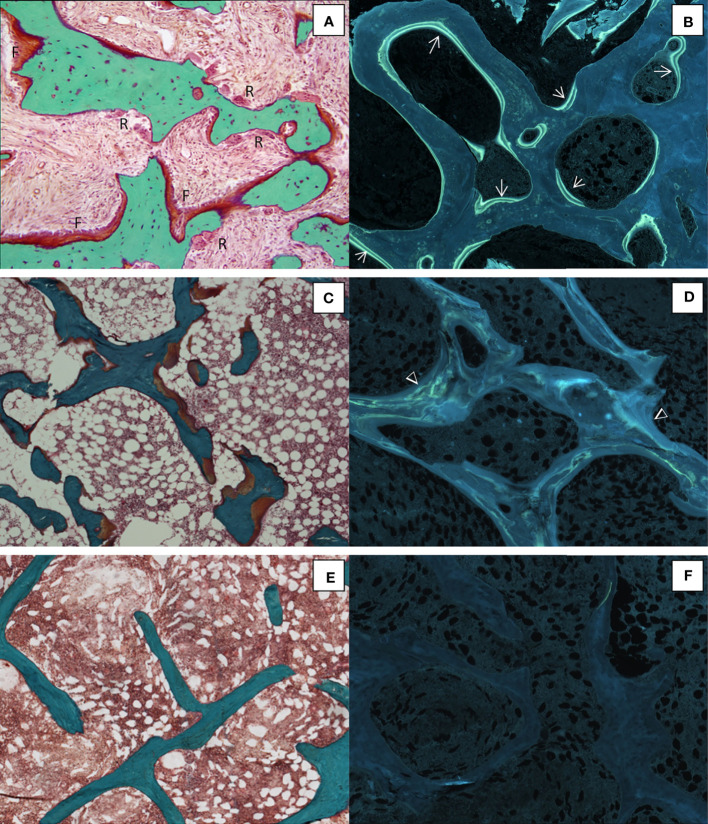
Different histological forms of renal osteodystrophy. **(A)** High bone remodeling with increased bone resorption (R) and formation (F) associated with numerous tetracycline labels (→) **(B)**; Osteomalacia with thick osteoid seams **(C)** and diffuse tetracycline labels (Δ) **(D)** ;Adynamic bone with no active bone surface **(E)** with no tetracycline label **(F)**. **(A**, **C**, **E)**, Goldner trichrome; **(B**, **D**, **F)**, unstained sections under ultraviolet light; x100.

Renal osteodystrophy is the bone manifestation of chronic kidney disease which may have different aspects. The first histological studies report that skeletal effects were both hyperparathyroidism and mineralization defects (148, 149). These bone abnormalities with the systemic disorders of mineral and bone metabolism are designed by the term Chronic Kidney Disease-Mineral and Bone Disorder (CKD-MBD). Biochemical markers of formation and resorption and parathyroid hormone levels may help to determine the type of CKD-MBD but cross-sectional studies have shown some discrepancies between biomarkers and bone histology. The bone biopsy has been recognized as the gold standard for the diagnosis and classification for renal osteodystrophy (150, 151) and is recommended when there are inconsistencies among biochemical parameters, unexplained fractures, severe vascular calcifications, a suspicion of aluminum toxicity, or before bisphosphonate treament (152). However, most nephrologists are reluctant to perform biopsies and the number of biopsies in renal insufficient patients remains insufficient. In these cases, the use of a smaller diameter trephine can provide information on the bone status. Renal osteodystrophy is classified according to the level of turnover, the mineralization, and the volume of bone (TMV classification). The predominant hyperparathyroid form is characterized by a marked increase in bone turnover with extended osteoid surface covered by osteoblasts, deep eroded surface with numerous osteoclasts, and an increase in labeled surfaces (**Figure 5A**). A fibrosis is often observed. The presence of thick osteoid seams with diffuse tetracycline labels signifies osteomalacia. In this latter form the turnover may be reduced (**Figure 5B**). Adynamic bone disease is characterized by a marked decrease of the bone turnover with absence of active bone formation or resorption and a low bone volume (**Figure 5C**). Mixed uremic osteodystrophy associates high turnover, mineralization defects and normal bone volume (152–154).

### Bone Mastocytosis

Systemic mast cell disease is associated with either osteoporosis or osteosclerosis with possible skin lesions. When biological analyses suggests a mastocytosis, the bone biopsy allows for the confirmation of an increased number of mast cells in the bone marrow. There has not been, however, a study that examined the sensitivity and specificity of the histologic analysis compared with tryptase measurement. An increased bone turnover is often reported with an unbalanced coupling between resorption and formation but without any mineralization defect (41, 155). Quantitative backscattered electron imaging showed a poorly mineralized bone in mastocytosis with osteosclerosis (156). The diagnosis is based on an increased number of mast cells in bone marrow, which may be close to the bone surface (**Figure 2C**). Mast cells are identified on bone sections stained with toluidine blue pH 2.6, May-Grünwald-Giemsa or acridine orange.

### Endocrine Diseases

Bone fragility related to an alteration of the bone remodeling exists in several endocrine diseases such as hyperthyroidism and Cushing’s syndrome (157). The high risk of fracture observed in acromegaly despite a normal BMD results from an alteration of the trabecular and cortical bone structure and architecture with a marked reduction of the osteoblasts number and activity (158).

## Bone Biopsy as a Research Tool

**Figure 6:**
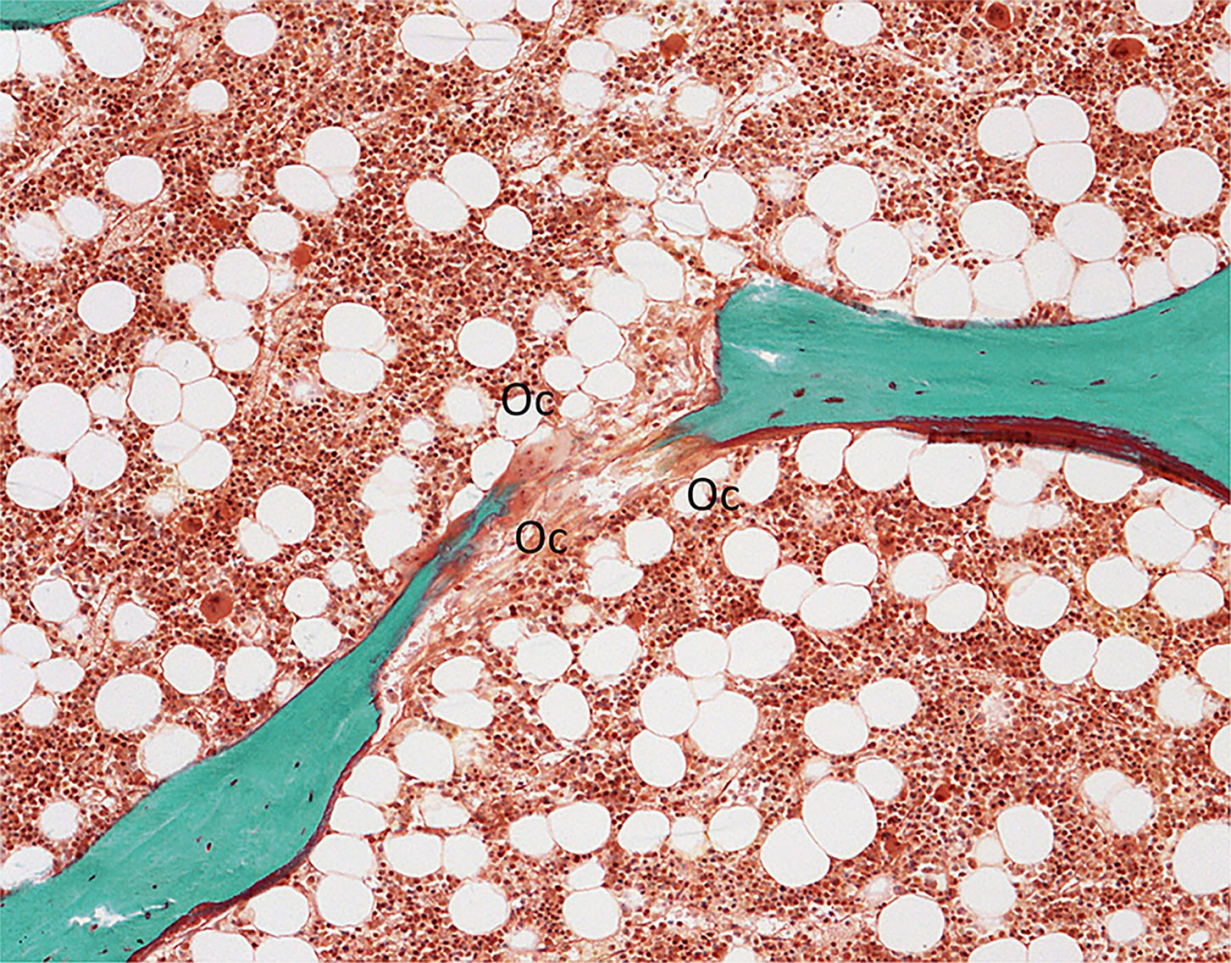
Trabecular perforation due to osteoclastic (Oc) resorption. Goldner trichrome; x100.

Initially, histomorphometric studies allowed for the characterization of bone diseases and osteoporosis appeared heterogeneous with different levels of remodeling (159, 160). The identification of high remodeling osteoporosis led to find endocrine disturbance such as primary hyperparathyoidism or hyperthyroidism (161). The analysis of bone biopsies also helped to understand the mechanism of remodeling, the coupling between resorption and formation, the process of mineralization, and thus, the pathogenesis of bone diseases (162). These studies showed that bone loss results from a negative balance at the bone structural unit, i.e., the amount of bone formed being lower than those previously resorbed, associated, or not with increased remodeling (163–165). The association of a high remodeling rate with deep resorption cavities produces a loss of trabecular plates. It may result in a trabecular perforation that has a major deleterious effect on the bone strength (166) (**Figure 6**). However, further studies showed that bone loss is not the only cause of bone fragility (52). The development of techniques that can be applied to bone sections has allowed the assessment of the other components of the bone quality. Besides the amount of bone and the microarchitecture, the material composition of bone (collagen and mineral) and the accumulation of microdamages contribute to the bone strength. In addition to bone histomorphometric analysis, bone biopsy can be used to assay collagen crosslinks. A decrease in bone turnover is associated with an accumulation of pentosidine (167) which is negatively correlated with bone strength (168–170). Bone material’s intrinsic properties can be analyzed by Fourier Transformed Infrared Microspectroscopy and the biomechanical behavior can be assessed by micro- or nano-indentation of bone samples (171, 172). All these components are controlled by the remodeling and any modifications of one of them result in bone fragility (157).

The coupling of osteoclasts and osteoblasts at the same remodeling site has been approched by the analysis of bone sections of hyperparathyroid patients showing that osteoclastic bone resorption and osteoblastic bone formation occur in a bone-remodeling compartments (BRC) (173). These authors observed that the canopy is separated from the bone marrow cavity by a monolayer of flat cells expressing osteoblast-like cell markers which is connected to capillaries. The contacts between these canopies and bone marrow capillaries initiate the bone remodeling (174).

During the past 30 years, basic and clinical studies have markedly enhanced the knowledge of the pathogenesis of bone diseases and especially on osteoporosis. The identification of molecules involved in the remodeling or the gene mutation in rare bone diseases have allowed for the development of new therapies (175). The magnitude of efficacy of new drugs is based on the fracture risk and bone mineral density but their approval requires a quantitative analysis of bone biopsies to assess their mechanisms of action at the tissue and cell level. Preclinical evaluations are firstly performed in animals, rodents, and large animals. However, especially in rodents, the histological procedures and the timing of double fluorochrome labeling must be adapted (176). Anti-osteoporotic treatments are divided in two main categories, inhibitors of the resorption and stimulators of bone formation.

**Figure 7:**
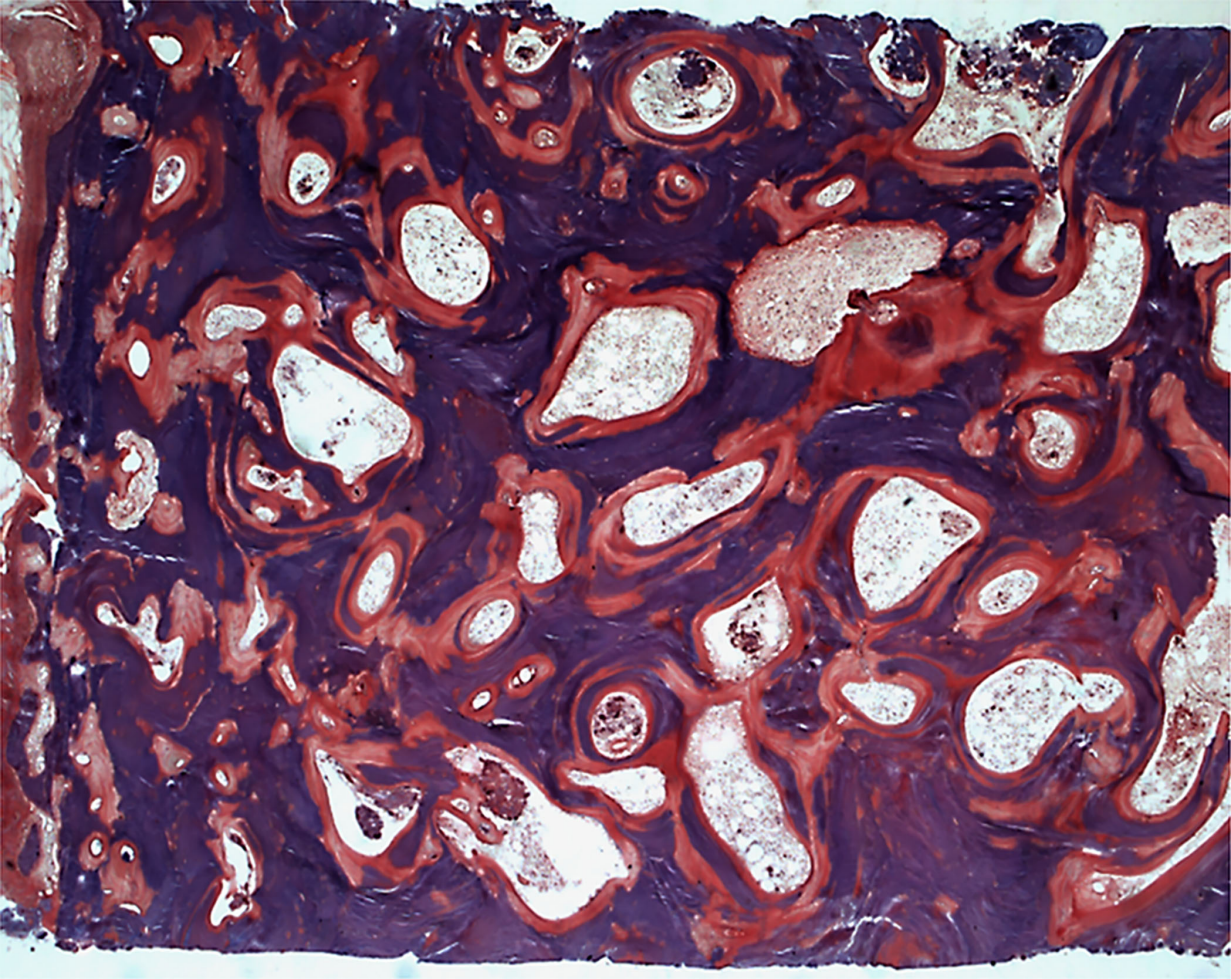
Skeletal fluorosis characterized by an osteosclerosis with increased osteoid surfaces and mineralization defects in the trabeculae and around the osteocyte lacunae. Solochrome cyanin R; x50.

Fluoride was one of the first treatments given in osteoporosis. High cumulative doses of fluoride induce a bone fluorosis which is characterized by a bone sclerosis due to an increased number of osteoblasts but with a toxic effect at the cell level (177) (**Figure 7**). Given at lower doses of 50 to 75 mg/day of sodium fluoride, it induced an increase in bone mass and osteoid parameters but also in mineralization defects (178–180). A marked decrease in trabecular bone quality was observed after long-term treatment (181). An abnormal mineral structure characterized by the presence of large crystals is present in newly formed bone which results in an increase in mineral density without improving the bone quality (182).

Bisphosphonates are synthetic analogues of pyrophosphate, with a phosphate-calcium-phosphate (PCP) bond. Many members of this class have been synthesized and divided into non-nitrogen-containing (etidronate, clodronate, tiludronate) and nitrogen-containing (pamidronate, alendronate, ibandronate, risedronate, zoledronate) bisphosphonates (183). Despite a different mechanism of action at the molecular level, and a different affinity to bone mineral, they strongly bind to hydroxyapatite crystals and inhibit the bone resorption (184). At the bone tissue level, the main effect of bisphosphonates is a marked decrease in the bone turnover. After 2 or 3 years, mineralizing surface and activation frequency decrease by 95% with alendronate (29), 80% with risedronate (185, 186), 73% with ibandronate (187), 91% with zoledronate (188) and 67% with pamidronate (189). Despite a marked decrease in the biochemical marker of bone resorption, no significant reduction in eroded surface was reported (29, 185, 187, 188) which may be explained by a prolonged reversal phase. Due to the coupling between resorption and formation, a marked decrease in bone formation is also observed with no evidence of an increase in bone mass despite a marked increase in bone mineral density measured by DEXA. All these observations suggest an increased secondary mineralization related to the low turnover (190, 191). In summary, the consequence of the inhibitory effect of bisphosphonates on bone resorption is a decreased remodeling which results in a reduced remodeling space, a preservation of the cancellous bone microarchitecture with no change in bone mass, and an increase in bone mineral density. All these effects contribute to improve the bone strength. The marked decrease in bone turnover with bisphosphonates has been suggested to inhibit the repair of microcracks and favor their accumulations (192). An increased microcrack density has been observed in dogs receiving high doses of bisphosphonates (193) but this has not been confirmed in humans (194).

Selective estrogen receptor modulators (SERM) are compounds which have estrogen agonist actions in some tissues and estrogen antagonist actions on other tissues. In postmenopausal women, raloxifene induces a trend in decreased eroded surface, bone formation rate, and activation frequency. These results suggest that the effects of raloxifene on bone tissue are similar to estrogen (195).

Strontium ranelate was initially presented as an agent with a dual mechanism of action able to stimulate the formation and reduce the resorption. This assumption was supported by *in vitro* studies (196, 197) and preclinical investigations in rodents (198). However, in postmenopausal osteoporotic women, strontium ranelate does not significantly modify the parameters of bone formation or resorption after 1 to 3 years of treatment (199). A second large clinical trial confirmed the absence of significant increase in bone formation and the decrease of bone resorption at the bone tissue level (200), despite a significant improvement of the bone mineral density and fracture rate. A recent study reports decreases of crystallinity and carbonate content and the possibility that strontium may create bounds with collagenous and non-collagenous proteins but does not modify the nanomechanical properties (201).

The anabolic effect of parathyroid hormone in osteoporotic patients was reported in the mid-1970s (202, 203). The effect of intermittent administration of teriparatide, the fragment 1-34 of parathyroid hormone, varies according to the study. No significant increase in mineralizing surface and bone formation rate after 18 months (204) or a decrease between 6 and 18 months (205) of treatment have been reported. In contrast, significant increases in the formation and resorption were observed after 28 days and 2 years (206). Beside a classical remodeling-based formation, a contribution of a modeling-based formation has been proposed with intermittent PTH (1–34) administration (204, 206). In these studies, the criterion to identify modeling surfaces was the presence of smooth cement lines. Instead of the creation of new formative sites without prior resorption, an extended bone formation beyond the limit of the scalloped reversal line onto the adjacent quiescent surface has been suggested, i.e., an overflow from the remodeling surface rather than *de novo* modeling (21, 207, 208). Abaloparatide binds to the RG subtype of parathyroid hormone type 1 receptor with a higher affinity than teriparatide. No major difference in both static and dynamic histomorphometric variables have been observed between placebo, teriparatide, and abaloparatide (209). Nevertheless, compared to teriparatide, serum bone formation marker (sP1NP) decreases after 3 months and the increase in the resorption marker (sCTX) was lower with abaloparatide (210).

Odanacatib, an investigational agent previously in development, is a cathepsin K inhibitor which reduces bone resorption *via* a mechanism distinct from other anti-resorptive drugs. Secretion of cathepsin K from the osteoclast results in degradation of type I collagen. The absence of cathepsin K activity in humans due to a CTSK mutation results in pycnodysostosis, a rare bone disease characterized by osteosclerosis and fractures (211, 212). Whereas other antiresorptive drugs decrease osteoclast activity or differentiation with a subsequent reduction in bone formation, odanacatib permits persistent osteoclast viability and activity and selectively inhibits the removal of matrix protein (213). At the bone tissue level, treatment with odanacatib did not change the dynamic parameters of bone formation and may increase the osteoclast number. An increased bone formation was observed at the periosteal surface contributing to the thickening of the cortices (214, 215). However, despite significant reduction of the fracture rate, odanacatib was ultimately withdrawn from the regulatory approval process after it was found to be associated with an increased risk of stroke.

Denosumab is a fully-human monoclonal antibody that binds receptor activator for nuclear factor κB ligand (RANKL), an essential factor for osteoclast differentiation and activity. Denosumab binds and reversibly inhibits the activity of RANKL and, therefore, the formation, activity, and survival of osteoclasts. As an antiresorptive agent, denosumab treatment induces a marked reduction in bone remodeling with both a decreased resorption and consequently, a diminution of formation. These effects observed after 2 and 3 years (216) are maintained after extension of the treatment up to 5 (217) and 10 (218) years. However, in contrast to bisphosphonates which remain incorporated in bone until resorption occurs, the effects of denosumab are reversible after discontinuation (219). The decreased bone turnover results in an elongation of the secondary mineralization and thus, an augmentation of the degree of mineralization (218). In addition, a reduction of the endocortical erosion depth with no change of the mean wall thickness also contributes to the greater gain of BMD with denosumab than with other antiresorptive agents (220). The persistence of a modeling-based formation process has also been suggested (221); when the remodeling is very low, modeling bone formation may sparsely occur to preserve the bone mass (222), especially in loaded regions (223). A transient increase in endogeneous PTH level after denosumab administration has been evoked (224) but not confirmed (225).

Romosozumab, a bone forming agent, is a humanized monoclonal antibody that binds and inhibits sclerotin, thereby, promoting osteoblast differentiation and activity. Sclerostin is a protein produced by osteocytes that inhibits the bone formation by inhibiting canonical Wnt signaling. Inherited sclerostin deficiency is characterized by a high bone mass (226). At the bone tissue level, romosozumab treatment induces an early and transient marked augmentation of bone formation parameters associated with a reduction of the resorption (22). This dual and opposite effect reflects a transient absence of coupling between resorption and formation. The occurence of a modeling-based bone formation, previously reported in animal studies (227, 228), has been confirmed in humans (229). However, a self-regulatory mechanism of the bone formation suggested by experimental studies (230) explains that later in treatment, only the decreased bone resorption remains associated with a decreased bone turnover as a consequence of the coupling. These effects result in an increased bone mass and improved microarchitecture. Romosozumab is the first anti-osteoporotic agent having both an anabolic and an antiresorptive action.

## Bone Histomorphometry in the Future

Computerized microscopy image analysis is widely used for diagnosis and prognosis in various fields of clinical practice and has improved the analysis of bone diseases. Recently, deep learning techniques have been developed (231) and applied in digital image processing (232), allowing for cell detection and classification (233). The application of these methods to bone histomorphometry will likely allow for easier diagnosis. The utility of machine learning based on biochemical testing, imaging, and clinical data has been reported in the diagnosis and fracture prediction in osteoporosis [see review (234)]. Combined with bone histomorphometry, the investigation of the molecular profile of circulating mesenchymal stem cells may provide information on the individual’s osteogenic potential (151)

Many questions remain regarding the mechanism of coupling, the role of the reversal phase, and the several weeks separating the osteoclastic resorption and the osteoblastic formation (235–237). Numerous factors released during the resorption phase have been identified as playing a role in the coupling between resorption and formation. These factors may be released from the resorbed bone matrix, secreted by osteoclasts or transported by microvesicles (238, 239). But the initiation of remodeling is not only based on the levels of these different factors but also requires a determinant for a specific site where osteoclasts and osteoblasts will successively work (240). A bone-remodeling compartment (BRC) separated from the bone marrow cavity by a monolayer of flat cells from the osteoblast lineage has been described (173). The extent of this canopy over remodeling sites varies in several pathological situations and a reduced canopy surface is associated with a smaller extent of bone-forming surfaces (173). Bone loss in postmenopausal osteoporosis is associated with absences of canopy above formation and eroded surfaces. Furthermore, an accumulation of arrested reversal surface and a decreased extent of surface of formation with a modification of osteoblasts morphology from a cuboidal to flattened shape is observed (241). A loss of BRC canopies is also reported in multiple myeloma (242), Cushing’syndrome (243), and glucocorticoid-induced osteoporosis (244). In recent years, additional observations have been provided by the Delaisse group (245, 246). At the beginning of the reversal period, mononuclear cells remove the demineralized collagen before the onset of osteoblasts and the secretion of the bone matrix. These cells have been initially identified as macrophages but ultrastructural analysis showed that they are bone lining cells able to activate matrix metaloproteinase (246). By analyzing 3D reconstruction of serial sections of Haversian systems in human long bone, these authors captured the events ranging from initiation of resorption to onset of formation as a functional continuum. They described a mixed “reversal-resorption” phase characterized by the presence of both osteoclasts and reversal/osteoprogenitor cells. The reversal/osteoprogenitor cells gradually matured into osteoblasts and bone formation is initiated only above a threshold cell density. The length of the reversal/resorption period depends on how fast osteoprogenitor recruitment reaches this threshold (247). The activation of remodeling is linked to the development of the vasculature close to the bone surface and this interaction between capillary and canopy provides osteoblasts progenitors (248). Based on these observations, an additional mechanism of alendronate has been recently reported where osteoprogenitors recruitment is slowed down on eroded surfaces and consequently, the onset of bone formation is delayed (249). These observations provide information to better understand how the balance between resorption and formation is regulated.

A better understanding of existing treatment is essential to improve their use. Over the past decades, studies have focused on the effects of new therapeutics for bone remodeling but forgot the process of modeling described more than 50 years ago (250). A modeling-based formation was firstly reported for teriparatide mainly due to an overflow of the remodeling surface (21). Denosumab strongly inhibits remodeling-based formation but little remaining modeling has been suggested (221). In contrast to other existing treatments, the early marked increase in bone formation with romosozumab results from a higher proportion of modeling than remodeling based formation (22, 229). Thus, from these observations, it appears that in addition to the remodeling, the modeling process may contribute to the antifracture efficacy of therapeutic agents and needs to be suitably assessed (251). Denosumab is an effective anti-osteoporotic agent but a rapid bone loss with an increased fracture risk is observed after treatment withdrawal, suggesting a rebound in osteoclast activity. McDonald et al. (252), using intra-vital imaging, observed a fission of osteoclasts into smaller motile cells named osteomorphs which were able to refuse and form osteoclasts in another site. Inhibition of RANKL by osteoprotegerin treatment results in the accumulation of osteomorphs able to rapidly fuse into active osteoclasts upon osteoprotegerin withdrawal. These observations may explain the rebound after denosumab discontinuation. These findings will open new investigations on the pathogenesis and treatment of bone diseases.

## Summary and Conclusion

The diagnosis of the majority of bone diseases is based on clinical, radiological, and biochemical examinations with the development of non-invasive methods as bone densitometry, biochemical markers, and quantitative computed tomography. Nevertheless, bone histomorphometry remains the only method for the study of bone at the tissue and cellular levels. Qualitative and quantitative analyses of transiliac bone biopsies require strict methodological conditions which have been initially described more than 60 years ago. Bone biopsy, which is an invasive method, has limited indicators for diagnostic purposes, especially in osteoporosis. The iliac crest being an unloading skeletal site, in contrast to the spine and the femoral neck which is prone to fracture is the only mean for diagnosing a mineralization defect. The recommended procedure is to collect a horizontal transiliac bone biopsy with a 7.5 mm inner diameter trephine. A previous fluorochrome double labeling is required to assess the mineralization and the dynamic of bone formation. For a better representation, sections are cut at 3 different levels and Goldner trichrome staining allows assessing both resorption and formation parameters. The progress of image analyzers, improved the analysis of bone sections, and the development of new image techniques will help the analysis of bone biopsy in the future. Bone histomophometry will continue to play a major role in the understanding of the pathophysiology of metabolic bone diseases and the evaluation of the safety and mechanisms of action of therapeutics.

## Author Contributions

PC and RC contributed to the manuscript and approved the final version.

## Conflict of Interest

The authors declare that the research was conducted in the absence of any commercial or financial relationships that could be construed as a potential conflict of interest.

## Publisher’s Note

All claims expressed in this article are solely those of the authors and do not necessarily represent those of their affiliated organizations, or those of the publisher, the editors and the reviewers. Any product that may be evaluated in this article, or claim that may be made by its manufacturer, is not guaranteed or endorsed by the publisher.
